# Ejaculatory and Orgasmic Dysfunction Following Prostate Cancer Therapy: Clinical Management

**DOI:** 10.3390/medsci7120109

**Published:** 2019-12-10

**Authors:** Travis P. Green, Jose Saavedra-Belaunde, Run Wang

**Affiliations:** 1 Division of Urology, Department of Surgery, University of Texas Health Science Center – McGovern Medical School at Houston, Houston, TX 77030, USA; Travis.P.Green@uth.tmc.edu (T.P.G.); tico2593@gmail.com (J.S.-B.); 2 MD Anderson Cancer Center, University of Texas, Houston, TX 77030, USA

**Keywords:** ejaculatory dysfunction, prostate cancer, dysorgasmia, climacturia, anejaculation, premature ejaculation, painful ejaculation, orgasm

## Abstract

The majority of sexual health research has focused on erectile dysfunction following prostate cancer treatment. Ejaculatory and orgasmic dysfunction are significant side effects following the treatment of prostate cancer. Orgasmic dysfunction covers a range of issues including premature ejaculation, anorgasmia, dysorgasmia, and climacturia. This review provides an overview of prevalence and management options to deal with orgasmic dysfunction. A Medline Pubmed search was used to identify articles relating to these problems. We found that orgasmic dysfunction has a very large impact on patients’ lives following prostate cancer treatment and there are ways for physicians to treat it. Management of patients’ sexual health should be focused not only on erectile dysfunction, but on orgasmic dysfunction as well in order to ensure a healthy sexual life for patients and their partners.

## 1. Introduction

Prostate cancer is currently the most common cancer found in men and has an annual prevalence of about 110 per 100,000 men per year in the United States. It is also the second most common cause of cancer death in men, responsible for approximately 19.2 deaths per 100,000 men in the United States [1]. The incidence of prostate cancer has steadily increased over the last decade and is expected to continue to rise [2]. Improvement in prostate cancer detection modalities such as prostate-specific antigen (PSA) have led to a younger patient population undergoing treatment [3]. The majority of prostate cancer is now discovered at clinical stage T1c [4]. Due to these advances, the 5-year survival rate for localized prostate cancer following treatment is 100% [1]. Therefore, it is important to study how treatment modalities may impact a patient’s life.

Standard treatment options for prostate cancer include radical prostatectomy, radiation therapy, and active surveillance [5]. The ProtecT has recently compared these treatment modalities and compared 10-year outcomes. Overall, 10-year prostate cancer-specific mortality was low and not significantly different between the arms [6]. However, these treatment modalities do contribute significantly to erectile, ejaculatory, and orgasmic dysfunction. Although most of the focus is on erectile dysfunction (ED), studies have shown that restoration of erectile function alone is not enough to achieve complete patient satisfaction [7].

Ejaculatory disorders can be subdivided into premature ejaculation (PE), delayed ejaculation/anorgasmia, unsatisfactory sensation of ejaculation (including painful ejaculation and ejaculatory anhedonia), and absent ejaculate [8]. In addition, there are other orgasmic complications including incontinence during sexual activity (climacturia). Studies have shown a 27–93% incidence of orgasmic dysfunction following radical prostatectomy (RP) [9,10]. In addition, radiation therapy has also been linked to orgasmic dysfunction. Anejaculation has been reported in 2–56% of patients and dissatisfaction in sex life has been seen in 25–60% of men after undergoing radiation therapy [11].

## 2. Definition of Orgasm

The International Consultation on Sexual Medicine described the experience of orgasm as a distinct cortical event, experienced phenomenologically, cognitively, and emotionally, associated with the perception of striated muscle contractions and resulting in semen expelled during ejaculation and mediated through sensory neurons in the pelvic region [12]. Orgasm continues to be the least understood phase of the sexual response system. Therefore, the data available for post prostatectomy and radiation orgasmic dysfunction are few. There is currently no orgasm-specific questionnaire to standardize the results [13].

## 3. Premature Ejaculation

Although there has been no correlation linked between premature ejaculation/orgasm and prostate cancer treatment, there are a significant number of men suffering from PE that have been diagnosed with prostate cancer. In 2016, Lin et. al. showed a prevalence of 63.7% of PE in 1202 men diagnosed with prostate cancer in Iran [14]. PE is the most common ejaculatory disorder and may present with first sexual encounter or be acquired later in life [15]. It is thought that the worldwide prevalence of PE has approached 30% [16].

Premature ejaculation has been described by DSM-V as a condition of short ejaculatory latency that causes personal distress and is beyond the patient’s ability to control. The patient must experience the disorder 75–100% of the time over the course of at least six months [17]. The American Urologic Association has echoed this statement by stating that premature ejaculation is ejaculation that occurs sooner than desired, either before or shortly after penetration, causing distress to either one or both partners [18]. The recommended ejaculatory latency time for diagnosing PE has varied in the literature from 1 to 2 min or sometimes less [19]. The diagnosis of premature ejaculation is based on a thorough sexual history. The specific etiology of PE is currently unknown and the current treatment options are not curative. Current treatment options are broken down into behavioral therapy, topical treatment, systemic therapy, and other therapies [20].

### 3.1. Behavioral Therapy

The two most common behavioral therapy techniques include the “stop-start” technique and the “squeeze” technique. The “start-stop” technique, developed by Semans in 1956, involves initiating intercourse and stimulating the penis until one has the urge to ejaculate. At this time, stimulation is avoided until the urge to ejaculate subsides [21]. This maneuver is repeated several times prior to allowing oneself to ejaculate. This works to improve the understanding of physical and emotional parameters in arousal [22]. The “squeeze” technique was developed by Masters and Johnson in 1970. This technique involves stimulating the penis until the urge to ejaculate appears and then squeezing the glans until this urge subsides [23]. Early studies showed up to a 97.8% success rate. However, more recent studies have been unable to replicate these results [24]. However, there have been studies showing an improvement in intravaginal ejaculation latency time (IELT) when behavioral therapy is used in conjunction with drug treatments as opposed to drug treatment alone [22]. Therefore, it appears beneficial to inform patients of these behavioral modifications and their role in the treatment of premature ejaculation.

### 3.2. Topical Anesthetics

It is believed that penile hypersensitivity may play an important role in the etiology of primary premature ejaculation [25]. There have been several studies performed showing that men with PE have a lower penile vibratory threshold, higher bulbocavernosus evoked potentials, and larger dorsal nerve somatosensory evoked potential amplitudes [25,26]. If patients with PE suffer from penile hypersensitivity, masking the sensory input from the penis using topical anesthetics could increase IELT [27]. Most commonly used topical anesthetics include lidocaine, benzocaine, prilocaine, and severance secret-cream. These local anesthetics work by stabilizing the membrane through inhibition of voltage-gated sodium channels. This works to decrease synaptic transmission and increase the threshold for ejaculation [28]. Combinations of these anesthetics also exist. One of the most popular and well-studied topical treatments includes a mixture of lidocaine and prilocaine. This cream contains 2.5% of both prilocaine and lidocaine and goes by the brand name of EMLA or AstraZeneca [29]. Multiple studies have suggested that this mixture works to increase a patient’s IELT and satisfaction of sexual performance [30,31]. In addition to creams, there exists aerosolized formulations of topical anesthetics. These aerosol sprays may be dissolved in a non-chlorofluorocarbon solution which will not penetrate fully keratinized skin. This allows the effect to be isolated to the glans penis and limits unwanted side effects [32].

### 3.3. Systemic Therapy

The etiology of PE is for the most part unknown. However, there have been studies to suggest that a contributing factor may be dysfunction of the 5-HT receptors [33]. It has been found that elevated levels of 5-HT have been found at many of the receptors involved with ejaculatory control. In addition, activation of the central 5-HT-mediated system has an inhibitory effect on ejaculation [34]. Therefore, it is believed that off-label use of selective serotonin reuptake inhibitors (SSRI), serotonin and norepinephrine reuptake inhibitors (SNRI), and tricyclic antidepressants (TCA) may work to improve PE through mediation of serotonin levels.

SSRIs are believed to induce delayed ejaculation by blocking presynaptic and somatodendritic serotonin transporters, and they enhance serotonergic neurotransmission and thereby activate 5-HT receptors [35]. Most patients are able to see the effects of SSRIs 1–2 weeks after initiating treatment [36]. However, some patients do see a decrease in effectiveness after 6–12 months of consistent use [36]. SSRIs currently used for the off-label treatment of PE include fluoxetine, paroxetine, citolapram, fluvoxamine and sertraline [33]. Side effects of SSRIs can include reduced libido, mild headache, nausea, sweating and dizziness [33].

SNRIs are most often used for treating psychiatric disorders or in chronic pain syndromes [37]. SNRIs work through reducing reuptake of both serotonin and norepinephrine in the synaptic cleft. This results in an increased concentration of both of these neurotransmitters [34]. One study, by Athanasios et al., showed that duloxetine, in comparison with placebo, increases IELT in patients with PE. They also showed that duloxetine was beneficial in increasing sexual desire and partner satisfaction [38]. Another study, performed by Ozcan et al., showed an improvement of IELT in addition to personal distress related to ejaculatory control in patients taking duloxetine for PE [39].

TCAs also play a role in management of PE. TCAs work to block the reuptake of both norepinephrine and serotonin and therefore are thought to work in a similar manner to SNRIs. In addition, TCAs have also been reported to block muscarinic, alpha-1 adrenergic, and histaminic receptors [40]. This has been shown to lead to a wide range of side effects including dizziness, confusion, difficulty urinating, and dry mouth [40]. In 1973, Eaton published the first study showing the effectiveness of TCAs in treating PE. This study reported “an almost 100% positive reaction” when treating PE with clomipramine [41]. Since this pioneering revelation, there have been multiple other studies showing a positive effect on sexual satisfaction when using clomipramine daily [41,42,43,44,45]. Clomipramine has been shown to have a stronger affinity for 5-HT transporters than other SSRIs and SNRIs. There has been one study showing a better increase in IELT with patients taking clomipramine when compared to sertraline and fluoxetine [46]. TCAs can be effective in treating PE, however the wide range of side effects make them a difficult choice [40].

Tramadol works as a centrally acting opioid analgesic normally used in the treatment of acute and chronic pain [47]. It has been shown to bind to central mu-opioid pain receptors and inhibit the reuptake of both serotonin and norepinephrine. It is thought that tramadol treats PE through the neuromodulation of serotonin and norepinephrine [48]. It has been shown by several studies to both increase IELT and sexual satisfaction in patients with PE [49,50,51]. Wu et al. performed a meta-analysis and systematic review to assess the safety and efficacy of tramadol in the treatment of PE. They were able to demonstrate an increase in IELT of 3 min when compared to placebo. However, there was no difference when comparing tramadol and paroxetine [52]. 

### 3.4. Other Therapies

There are other alternative therapies for treatment of PE, however the results are varied. One therapy that has been explored is acupuncture. One systematic review showed that there have only been three randomized control trials showing acupuncture to be more effective than placebo in treating PE [53]. However, acupuncture has not been shown to be as effective when compared to paroxetine alone [54]. Yoga has also been studied in the treatment of PE with mixed results. It has been theorized that yoga is helpful in promoting body awareness, but many trials have shown no improvement in PE over placebo [55]. However, one prospective study did show a significant increase in IELT [56]. An overview of common medical therapies for premature ejaculation is found in Table 1. 

## 4. Anorgasmia/Anejaculation

Both radiation therapy and radical prostatectomy can alter a patient’s ability to achieve orgasm or ejaculation. After external beam radiation therapy, a lack of ejaculation was reported in up to 89% of patients [57,58,59]. This has also been associated in a dissatisfaction with a patient’s sex life in 25–60% of patients after radiation therapy [11]. Additionally, there has been a reported decrease in intensity of orgasm associated with radiation therapy [58]. In contrast, after radical prostatectomy, anejaculation is expected as the prostate and seminal vesicles are removed, the vas deferens is disconnected, and the urethra is altered during the surgery [9]. However, it has been reported that more than 60% of patients were unaware that they would not expel semen following RP [60]. The manipulation during surgery also may lead to alterations in orgasm and anorgasmia. Anorgasmia and altered sensation during orgasm has been reported in up to 78% of patients following RP [61]. Anorgasmia and anejaculation are common side effects following prostate cancer treatment and patients must be counseled on this risk prior to undergoing treatment.

One large study performed by Hollenbeck et al. prospectively looked at the sexual health outcomes of 671 men up to 52 months following RP and compared them with age matched control patients. This study found that age, nerve-sparing technique, and prostate size all contributed to changes in sexual health after RP. Of patients younger than 58 years who suffered from anorgasmia, 16% underwent bilateral nerve-sparing RP, 32% underwent unilateral nerve-sparing RP, and 33% underwent non-nerve-sparing RP. This is compared to a rate of only 6% in the control group. In men older than 69 years, 42% underwent bilateral nerve-sparing RP, 42% underwent unilateral nerve-sparing RP, and 70% underwent non-nerve-sparing RP. In this age group, the control group had a rate of anorgasmia in 33% of men [62]. Another study, performed by Dubbelman et al., reported that 33.2% of 458 patients had anorgasmia at a mean follow-up of 23.6 months after RP. They mirrored the study by Hollenbeck, showing a significant correlation between anorgasmia and older age and nerve-sparing status [63]. These findings were again confirmed by Tewari et al., who reported an anorgasmia rate of 14% at a mean of 36 months after RP in 408 patients. This study again found a significant correlation with age and nerve-sparing status [64]. 

Although several studies have demonstrated the correlation between prostate cancer treatment and anorgasmia, the exact cause is not well known. Some have theorized that anorgasmia may be considered a psychological event after prostate cancer treatment [10]. Others have postulated that the correlation between orgasmic function, post-operative erectile function, and nerve-sparing technique may imply that neurapraxia could play a role [64]. Another theory is that anorgasmia is directly correlated with erectile dysfunction. In this setting, it is thought that ED may limit the stimulation of the dorsal penile nerve and therefore decrease the intensity of orgasm [64]. In addition, with the decrease in ejaculate and inability to feel prostatic or seminal vesical contractions following prostate cancer treatment, the sensation of orgasm may be less satisfying. In this respect, Messaudi et al. showed that after RP, 54% of patients were bothered by their anejaculation and 8.2% avoided intercourse for this reason alone [61]. 

Anorgasmia or delayed orgasm may lead to significant distress in patients’ lives following prostate cancer treatment. Unfortunately, there is currently a lack of effective pharmacotherapies for this condition. There are currently no FDA approved pharmacotherapies for anorgasmia or delayed orgasm. The first consideration should be to discontinue any medications, such as SSRIs, that could be contributing. It is good practice to work with the physician prescribing the medication and have them coordinate a drug holiday, cessation, or substitution [65]. Cabergoline, a dopamine agonist, has been used off-label in the treatment of anorgasmia. This pharmacotherapy works to reduce serum prolactin levels. Corona et al. have shown that prolactin levels progressively increased from patients with severe PE towards those with anejaculation [66]. Hollander et al. published a non-randomized, non-placebo-controlled study in which they administered cabergoline to potentially improve orgasmic sensation in men. In this study, 131 men, of which 23 (17.6%) had undergone RP, were given cabergoline for a median duration of 9.8 months. They showed that 66.4% of patients responded to this treatment. In addition, there was no correlation between RP and response to treatment [67]. Phosphodiesterase type 5 inhibitors have also been proposed as a treatment for orgasmic dysfunction following nerve-sparing RP. Nehra et al. performed a randomized, placebo-controlled, double-blinded trial which showed significant improvement in orgasmic function with a 3-month course of vardenafil. This study quantified orgasmic function using the International Index of Erectile Function [68]. Although this study was promising, other studies have failed to show statistically significant improvement in orgasmic function through the use of phosphodiesterase 5 inhibitors [69].

## 5. Climacturia

Climacturia, also known as orgasm-associated urinary incontinence, is defined as the involuntary loss of urine at the time of orgasm. True rates of climacturia following treatment for prostate cancer therapy are currently not well known [70]. Clavell et al. published a recent review article on post RP orgasmic dysfunction and found that prevalence was in the range of 20–93%. However, they determined that the average prevalence of climacturia following RP was closer to 30% [10,61,71,72,73,74,75,76,77,78]. O’Neill et al. published a retrospective study to determine rates of climacturia for patients undergoing either radiation or radical prostatectomy. In the 412 patients surveyed, they found climacturia to be present in 5.2% of post radiation patients and 28.3% in post RP [74]. Lee et al. have published the “significant bother” for patients suffering from climacturia to be as high as 47% [71]. Although rates of climacturia following prostate cancer therapy are significant, Choi et al. were able to show that time since surgery is an independent predictor of climacturia after RP [73]. Expanding on this finding, Capogrosso et al. reported a clear trend towards recovery in climacturia patients following RP. In a prospective study of 749 patients, they showed 24% of patients recovering at 84 months vs. 2.3% and 5.5% recovering at 12 and 24 months, respectively. This study also found that robot-assisted laparoscopic surgery is an independent predictor of faster recovery of climacturia [70]. These studies show that although climacturia rates are significant following prostate cancer treatment, these patients often improve over time. No differences in the rate of climacturia have been found based on age, preoperative erectile function, nerve-sparing status, and, as mentioned earlier, daytime incontinence [71].

The mechanism for climacturia following prostate cancer treatment has not yet been proven. Some have hypothesized that the removal of the internal urethral sphincter in conjunction with relaxation of the external urethral sphincter during orgasm may contribute [72]. However, there has not yet been a clear association discovered between daytime incontinence and climacturia. Therefore, we can assume that climacturia is not simply an extension of post treatment urinary incontinence [71,73]. It has also been thought that loss of urethral length following RP may contribute to climacturia. Choi et al. showed loss of penile length to be an independent predictor of climacturia [73]. This was confirmed by Manassero et al. who performed video urodynamic evaluation on patients with climacturia who were otherwise continent following RP. This study showed a significantly shorter urethral length in patients with climacturia [79].

Climacturia can have negative effects on a patient’s sexual health and unfortunately a definitive treatment does not exist. However, there are several lifestyle modifications, pharmacotherapies, and surgical interventions that may be of benefit. Some have suggested that patients should use different coping strategies including such as emptying the bladder prior to intercourse and use of condoms [71]. Pelvic floor therapy has shown some promise in a few very small trials. Sighinolfi et al. evaluated 3 patients with stress urinary incontinence and climacturia 12 to 18 months following RP. These patients were started on a pelvic floor rehabilitation program including active pelvic floor muscle exercises, electromyographic biofeedback for strength and endurance, and electrical stimulation. There was found to be a lower rate of climacturia in all 3 patients following 4 months of treatment. Garaerts et al. mirrored these findings in a randomized control trial of 33 men using pelvic floor muscle training (PFMT) 12 to 15 months following RP. They showed a significant improvement in climacturia after 3 months of PFMT [80]. Variable tension loops used for augmentation of transurethral alprostadil suppositories have been studied as a non-invasive option for climacturia. Mehta et al. included 24 patients who reported the degree of pre-treatment climacturia as small, medium, and large in 16%, 72%, and 12% of patients, respectively. After using the variable tension loop, degree of climacturia was reported as 28%, 26%, 0%, respectively [81]. Another alternative to surgical correction of climacturia was presented by Fitzpatrick and Bella. They reported use of a soft silicone occlusion loop in a 5-patient cohort of patients with climacturia who had previously failed using condoms and penile variable tension loops and were not interested in surgical management. They reported improvement in all 5 patients and complete resolution in 4 [82]. 

As a more invasive option, Jain et al. evaluated placement of an artificial urinary sphincter or a male urethral sling in a small cohort of 11 patients. These patients had urinary incontinence and climacturia at a mean of 33.5 months following RP. All 11 patients reported an improvement in climacturia [83]. Andrianne and Van Renterghem recently presented a new surgical procedure called the “mini-jupette” mini sling. The procedure uses a piece of poly-propylene mesh placed at the time of inflatable penile prosthesis insertion. This mesh is anchored to the medial portion of the bilateral corporotomy sutures. This causes the mesh to compress the bulbar urethra when the implant is inflated [84]. Yafi et al. looked at 38 patients with ED, of which 30 had climacturia who were undergoing IPP placement with concomitant placement of mini-jupette graft. After a mean follow-up of 5.1 months, 78.6% of patients had improvement in their climacturia when queried about episode frequency, and 68% showed complete resolution [85]. Although these studies report on relatively small cohorts of patients, these procedures do have a promising future in the treatment of climacturia.

## 6. Painful Orgasm/Dysorgasmia

Dysorgasmia, defined as painful orgasm, has a prevalence of 3.2–18% following RP [10]. A similar prevalence of 3–11% has been reported in patients following radiation therapy for prostate cancer [11]. Painful sensations have been reported at the level of the penis, rectum, testicles, or lower abdomen [76]. In a series of 702 patients who underwent RP, Matsushita et al. reported the rate of dysorgasmia to be 12%. They also noted that 70% of patients felt pain in the penis and 22% felt the sensation in the testes [86]. Although the rate of dysorgasmia following treatment is significant, it has been reported that the prevalence and severity decrease over time. Capogrosso et al. reported recovery rates of 10% and 30% at 12 and 60 months following RP, respectively. Surgical technique may also play a role in prevalence of dysorgasmia. This same study showed that dysorgasmia rates following open RP were 11.6% compared to 7.1% following robotic assisted RP [70].

Treatment for dysorgasmia is limited. This could be partially caused by our poor understanding behind the etiology of this disorder. Barnas et al. theorized that dysorgasmia could be a result of closure of the bladder neck leading to spasms of the vesicourethral anastomosis and or pelvic floor musculature dystonia following RP [76]. Another study by Barnas et al. looked at the possibility of treating dysorgasmia with tamsulosin 0.4 mg daily. The study looked at 98 patients who received this medication for at least 4 weeks. Of patients treated with tamsulosin, 77% reported significant improvement in pain and 12% noted complete resolution of their pain. This data supports the hypothesis that the pain could be related to bladder neck and/or pelvic floor spasms [87]. There have been other studies using α-blockers for dysorgasmia which have not shown a significant impact in symptoms [88].

## 7. Discussion

Orgasm is defined as a distinct cortical event, experienced phenomenologically, cognitively, and emotionally, associated with the perception of striated muscle contractions and resulting in semen expelled during ejaculation and mediated through sensory neurons in the pelvic region [12]. This review has summarized some of the orgasm-associated problems that men experience following treatment of prostate cancer. Although some factors such as older age, higher body mass index, and poor orgasmic or erectile function predict a worse outcome in orgasmic function following treatment, there still remains a substantial prevalence of these disorders in all patients [89]. Sexual function remains an important aspect of a patient’s quality of life [90]. Satisfaction of orgasm is correlated with marital satisfaction, stability and happiness [91]. It remains difficult to assess post-prostate cancer treatment orgasmic dysfunction as there is currently no validated questionnaire. Most of the studies that were discussed in this review used the non-validated International Index of Erectile Function which contains only two questions regarding orgasmic dysfunction. More work needs to be done to develop a validated questionnaire and provide a standardized way to explore orgasmic dysfunction.

Currently, many aspects of orgasmic dysfunction can be very difficult to treat. Physicians should begin counseling patients and setting expectations prior to the treatment of prostate cancer in order to avoid disappointment. It has been shown that 60% of patients following radical prostatectomy did not know that anejaculation was expected [60]. Physicians should touch on the difficulty of reproduction following prostate cancer treatment. Sperm banking prior to treatment should always be discussed due to the known anejaculation following prostatectomy and high prevalence after radiation. Otherwise, testicular sperm extraction may be required following their treatment course [59]. Sexual recovery should not only focus on erectile dysfunction but orgasmic dysfunction as well. It remains important for physicians to stress that patients should continue to pursue sexual activity and there are options for orgasmic dysfunction.

## Figures and Tables

**Table 1:** Summary of off-label treatments for premature ejaculation.

Drug	Description	Side Effects
SSRI (dapoxetine, citalopram, fluoxetine, fluvoxamine, paroxetine, sertraline)	Activation of 5-HT receptors have inhibitory effects on ejaculation pathway	Reduced libido, mild headache, nausea, sweating and dizziness
SNRI	Increase 5-HT and norepinephrine in synaptic cleft resulting in inhibition of ejaculation pathway	Reduced libido, mild headache, nausea, sweating and dizziness
TCA (clomipramine)	Block the reuptake of both norepinephrine and serotonin and therefore are thought to work in a similar manner to SNRIs	dizziness, confusion, difficulty urinating, and dry mouth
Tramadol	Neuromodulation of central 5-HT and norepinephrine	Potential for abuse and dependency, constipation, dizziness, nausea, vertigo, headache
Topical Anesthetic	Decrease hypersensitivity to penile stimulation	Burning, erythema, changes in skin color
Behavioral Therapy	Improved control over orgasm response	minimal

SSRI: selective serotonin reuptake inhibitor; SNRI: serotonin-norepinephrine reuptake Inhibitor; TCA: tricyclic antidepressant; 5-HT: 5-hydroxytryptamine.
